# Ferulic Acid Improves Depressive-Like Behavior in Prenatally-Stressed Offspring Rats via Anti-Inflammatory Activity and HPA Axis

**DOI:** 10.3390/ijms20030493

**Published:** 2019-01-24

**Authors:** Xingxing Zheng, Ying Cheng, Yiwei Chen, Yisong Yue, Yingchun Li, Sizhe Xia, Yang Li, Huanhuan Deng, Junli Zhang, Yanjun Cao

**Affiliations:** 1 Key Laboratory of Resource Biology and Biotechnology in Western China (Northwest University), Ministry of Education, Xi’an 86-710069, China; zheng@stumail.nwu.edu.cn (X.Z.); 15829758592@163.com (Y.C.); 2016113142@stumail.nwu.edu.cn (Y.C.); yueyeson123@gmail.com (Y.Y.); 201831945@stumail.nwu.edu.cn (Y.L.); xiasizhe@stumail.nwu.edu.cn (S.X.); 2017113115@stumail.nwu.edu.cn (Y.L.); m17782526319@163.com (H.D.); 15191905972@163.com (J.Z.); 2 Shaanxi Province Biomedicine Key Laboratory, College of Life Sciences, Northwest University, Xi’an 710069, China

**Keywords:** Ferulic Acid, Prenatal stress, Depression, NF-κB, glucocorticord receptor, Inflammatory cytokines

## Abstract

Prenatal stress (PS) can increase the risk of nervous, endocrine and metabolic diseases, and immune dysfunction. Ferulic acid (FA) is a dietary phenolic acid that has pharmacological properties, including potent anti-inflammatory action. We used male, prenatally-stressed offspring rats to investigate the anti-depressive-like effects and possible anti-inflammatory mechanism of FA. We determined the animal behaviors, and the mRNA expression and concentration of inflammatory cytokines, and HPA axis. In addition, we assessed the modulation of hippocampal nuclear factor-κB (NF-κB) activation, neuronal nitric oxide synthase (nNOS) and glucocorticoid receptors (GR) expression via western blotting and immunohistochemistry. Administration of FA (12.5, 25, and 50 mg/kg/day, i.g.) for 28 days markedly increased sucrose intake, and decreased immobility time and total number of crossings, center crossings, rearing, and grooming in the male PS offspring. FA significantly reduced IL-6, IL-1β, and TNF-α concentration and increased IL-10 concentration in male, prenatally-stressed offspring, stimulated by the NF-κB pathway. In addition, FA inhibited interleukin-6 (*IL-6*), interleukin-1β (IL-1β), and tumor necrosis factor-α (TNF-α), and increased *interleukin-10 (IL-10)* mRNA and protein expression. Furthermore, FA markedly decreased the serum adrenocorticotropin (ACTH) and corticosterone concentration by the increase of GR protein expression. Taken together, this study revealed that FA has anti-depressive-like effects in male, prenatally-stressed offspring, partially due to its anti-inflammatory activity and hypothalamic-pituitary-adrenal (HPA) axis.

## 1. Introduction

Prenatal stress (PS), which refers to stress during pregnancy, has been reported to exert a wide variety of negative emotional and behavioral effects on both human and animal offspring, including depression, anxiety, attention deficit hyperactivity disorder, and especially learning and memory deficits [1,2,3]. PS increases reactivity of the hypothalamic–pituitary–adrenal (HPA) axis in rats [4,5], which may affect immune responses. Immune cells express receptors for a variety of hormones [6] and glucocorticoids (GCs) can affect the development and function of the immune system [7,8].

Depression is a common mental disorder with symptoms including loss of interest or pleasure (anhedonia), a depressed mood, decreased energy, disturbed sleep or appetite, poor concentration and feelings of guilt or low self-worth [9]. There is no direct neural connection between the mother and fetus, the alterations of neuronal morphology induced by maternal stress are produced by the action of hormones at crucial periods of development. Prolonged GC exposure of the mother can reduce the number of hippocampal neurons of the offspring [10]. The hippocampus is particularly vulnerable to the effects of GCs because of the highest density of glucocorticoid receptor (GR) within the brain, which is intrinsically linked to both learning and memory and emotion. It has been reported that PS increases the HPA axis reactivity and circulating levels of the stress hormones GCs, and decreases the glucocorticoids receptor (GR) protein expression [11]. Therefore, reduction in serum corticosterone level may contribute to protecting the hippocampus neurons and improve depressive behavior. For the immature immune system of intrauterine fetus, elevated GCs can reduce the ability of self-protection, especially in terms of cellular immunity [6]. GCs can influence the process of maturation and growth of leukocyte, and regulate many cytokines [8,12]. Study has shown that inflammation plays a pivotal role in the pathogenesis of depression [9]. Animal models of depressive disorders have shown an increase in pro-inflammatory markers, such as interleukin-6 (IL-6), interleukin-1β (IL-1β) and tumor necrosis factor alpha (TNF-α). In addition, IL-1β, IL-6, and TNF-α mRNA expression are increased in the cortex, hypothalamus, and hippocampus of stressed rats [13,14]. Nuclear factor (NF)-κB is a pro-inflammatory transcription factor and a major mediator of inflammatory pathways, and regulated many cytokines, such as TNF-α, IL-6, IL-1, and IL-10. Many antidepressants have been shown to suppress NF-κB activation and pro-inflammatory transcription [15,16,17,18].

In recent years, Chinese traditional medicine has begun to focus more on the treatment of depression. Ferulic acid (FA) (4-hydroxy-3-methoxycinnamic acid), is a phenolic acid which is present in many plants such as *Ferula teterrima* Kar. Et Kir., *Angelica sinensis*, *Cimicifuga foetida* L., and *Ligusticum chuanxiong* Hort. It has a variety of biological effects including anti-inflammatory, anti-epileptogenic, anticancer and antioxidant activities [19,20]. In particular, a large number of animal experiments also show that FA can reverse memory loss in mice caused by inflammation, elevate the carbonyl protein level and reduce nerve cell injury [20,21,22,23]. In our previous studies, we focused on the negative effect of PS on the offspring and the specific mechanism, including the impaired GR and increased HPA axis reactivity. Taken together, these studies suggest that FA improves the depression induced by stress, but the effect and underlying mechanisms remain unclear. Therefore, these findings compelled us to explore whether FA had an effect on improving depression induced by PS in offspring.

The major objective of this study was to investigate the possible anti-depressive and anti-inflammatory effects of FA in prenatally-stressed offspring, and its possible mechanism. After 28 days of FA treatment in prenatally-stressed offspring rats, we measured the behavioral tests, including the sucrose preference test, forced swimming test (FST) and open field test (OFT). Then we detected the concentration and the expression of mRNA of TNF-α, IL-6, IL-10 and IL-1β in the hippocampus. We also measured the serum adrenocorticotropin (ACTH) and corticosterone levels, and evaluated the protein expression of NF-kB and GR in the hippocampus.

## 2. Results

### 2.1. Effects of FA on Sucrose Preference Test

**Figure 1:**
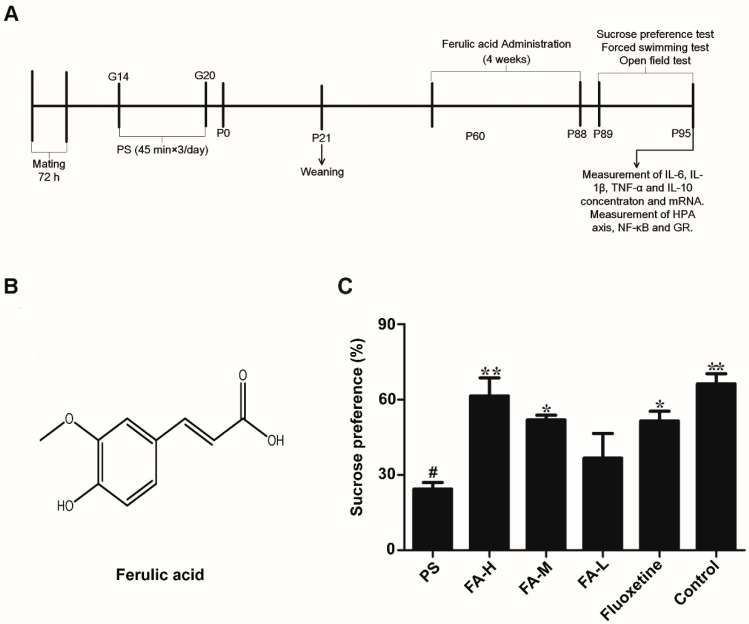
Chemical structure and effects of ferulic acid (FA) on the sucrose preference in offspring rats exposed to prenatal stress. (**A**) Timeline showing a summary of the experimental design. G: gestational age; P: postnatal age (days). PS: prenatal stress; (**B**) Chemical structure of FA; (**C**) Effects of FA on the sucrose preference. The percentage of sucrose consumed at sucrose concentrations of 2%. Values represent means ± SEM (*n* = 8). * *p* < 0.05 and ** *p* < 0.01 compared with PS group, ^#^ *p* < 0.05 compared with Control group. FA-L: FA at low 12.5 mg/kg/day, FA-M: FA at medium 25 mg/kg/day, FA-H: FA at high 50 mg/kg/day.

There was a significant reduction in the percentage of sucrose consumed in PS rats when compared with the control group respectively (*p* < 0.05; Figure 1). Moreover, when FA-L, FA-M, FA-H, or fluoxetine were administrated for 28 days, there was a significant increase in sucrose preference compared with the PS group (*p* < 0.05; Figure 1).

### 2.2. Effects of FA on Immobility in the Forced Swim Test

**Figure 2:**
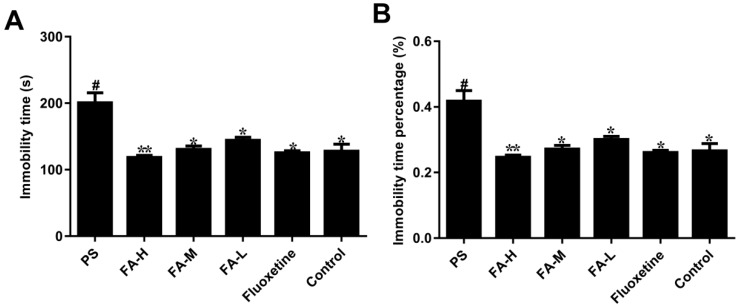
Effects of ferulic acid (FA) on the forced swimming test of offspring rats exposed to prenatal stress. (**A**) Immobility time in forced swimming test. (**B**) Immobility time percentage in forced swimming test. Values represent means ± SEM (*n* = 8). * *p* < 0.05 and ** *p* < 0.01 compared with PS group, ^#^ *p* < 0.05 compared with Control group. FA-L: FA at low 12.5 mg/kg/day, FA-M: FA at medium 25 mg/kg/day, FA-H: FA at high 50 mg/kg/day.

The result of FST is presented in Figure 2. After PS treatment, immobility time (*p* < 0.05, Figure 2A) and the immobility time percentage (*p* < 0.05, Figure 2B) in the male offspring were significantly increased compared with control group respectively. Meanwhile, following the administration of FA-L, FA-M, FA-H or fluoxetine for 28 days, the immobility time (*p* < 0.05, Figure 2A) and the immobility time percentage (*p* < 0.05, Figure 2B) were remarkably decreased in the male offspring.

### 2.3. Effects of FA on Open Field Test

**Figure 3:**
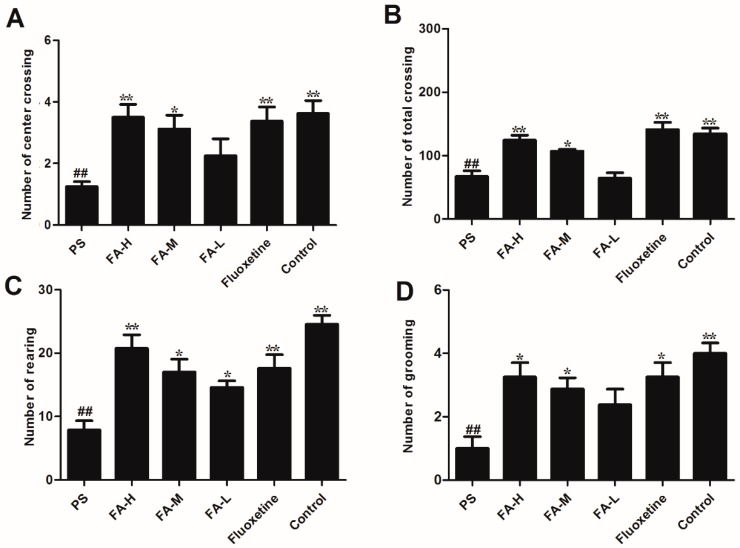
Effects of ferulic acid (FA) on the open field test of offspring rats exposed to prenatal stress. (**A**) The number of total crossings. (**B**) The number of center crossings. (**C**) The number of rearing. (**D**) The number of grooming. Values represent means ± SEM (*n* = 8). * *p* < 0.05 and ** *p* < 0.01 compared with PS group, ^##^ *p* < 0.01 compared with Control group. FA-L: FA at low 12.5 mg/kg/day, FA-M: FA at medium 25 mg/kg/day, FA-H: FA at high 50 mg/kg/day; Flx: fluoxetine.

As shown in Figure 3, there was a reduction in the number of center crossings (*p* < 0.05), total crossings (*p* < 0.05), rearing (*p* < 0.05), and grooming (*p* < 0.05) in PS offspring rats when compared with control rats. Treatment with FA significantly increased the PS-induced reduction (*p* < 0.05).

### 2.4. Effects of FA on ACTH and Corticosterone Levels

**Figure 4:**
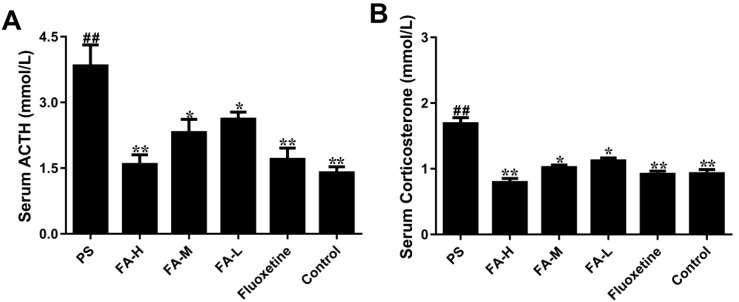
Effects of ferulic acid (FA) on the serum adrenocorticotropin (ACTH) and corticosterone levels of offspring rats exposed to prenatal stress. (**A**) Serum ACTH levels. (**B**) Serum corticosterone levels. Values represent means ± SEM (*n* = 8). * *p* < 0.05 and ** *p* < 0.01 compared with PS group, ^##^ *p* < 0.01 compared with Control group. FA-L: FA at low 12.5 mg/kg/day, FA-M: FA at medium 25 mg/kg/day, FA-H: FA at high 50 mg/kg/day.

To test if the protective role of FA in depressive-like behavior induced by PS is achieved by reducing the levels of serum ACTH and corticosterone, we assessed the effects of FA on serum ACTH and corticosterone levels in offspring rats exposed to PS (Figure 4). There was a significant interaction (*p* < 0.01), with effects for PS offspring tars (*p* < 0.01) and difference in FA treatment (*p* < 0.05) with serum ACTH and corticosterone levels. FA significantly reversed the elevated serum ACTH and corticosterone levels in offspring rats exposed to PS. Interestingly, fluoxetine treatment markedly prevented PS-induced offspring changes higher than those in the control group in serum ACTH and corticosterone levels. These results indicate that FA improves the depressive-like behavior by affecting the levels of serum ACTH and corticosterone.

### 2.5. Effects of FA on Nissl Staining in the Hippocampus of Offspring

**Figure 5:**
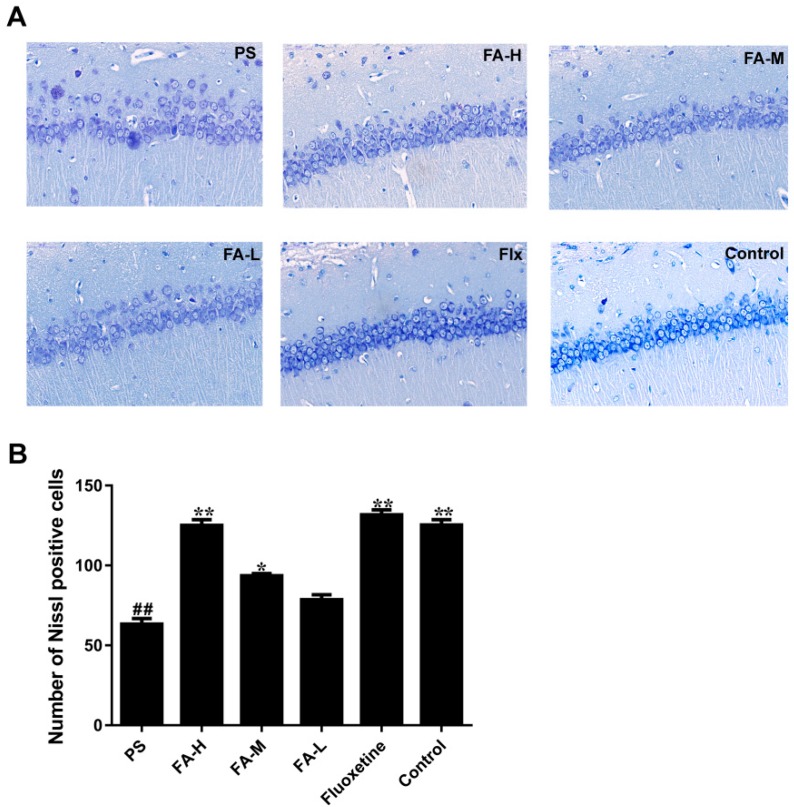
Effect of ferulic acid (FA) on hippocampus CA3 region neuron density in offspring rats exposed to prenatal stress. (**A**) Nissl staining of hippocampus CA3 region sections (magnification 400×, Scale bar = 50 μm), and (**B**) quantification of Nissl bodies in the hippocampus CA3 region. Data were expressed as mean ± SEM. *n* = 3 in each group, indicating at least three independent experiments in each animal. * *p* < 0.05 and ** *p* < 0.01 compared with PS group, ^##^ *p* < 0.01 compared with Control group. FA-L: FA at low 12.5 mg/kg/day, FA-M: FA at medium 25 mg/kg/day, FA-H: FA at high 50 mg/kg/day; Flx: fluoxetine.

Typical neuropathological changes, including neuron atrophy and nucleus shrinkage, were observed by Nissl staining in the CA3 of hippocampus in PS group, and these neuronal damages were ameliorated after the treatment of FA (*p* < 0.05, Figure 5).

### 2.6. Effects of FA on Neuronal NOS-Positive Expression in the Hippocampus of Offspring

**Figure 6:**
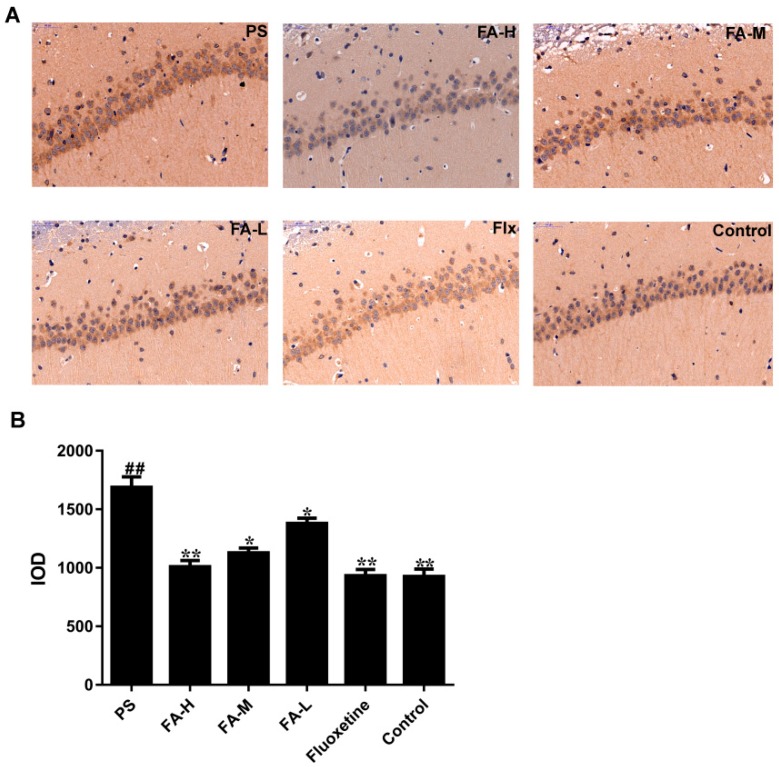
Effects of ferulic acid (FA) on the neuronal nitric oxide synthase (nNOS)-positive expression in hippocampal CA3 region of offspring rats exposed to prenatal stress. (**A**) nNOS expression was examined by immunohistochemistry (magnification 400×, Scale bar = 50 μm), and (**B**) Bar graph showing semi-quantitative analysis of nNOS via quantified integrated optical density (IOD). Values represent means ± SEM (*n* = 3). * *p* < 0.05 and ** *p* < 0.01 compared with PS group, ^##^ *p* < 0.01 compared with Control group. FA-L: FA at low 12.5 mg/kg/day, FA-M: FA at medium 25 mg/kg/day, FA-H: FA at high 50 mg/kg/day; Flx: fluoxetine.

A high expression of neuronal nitric oxide synthase (nNOS) in the PS group was detected by immunohistochemical analysis (*p* < 0.05 vs. control group) (Figure 6), which indicated that PS induced inflammation in the CA3 region of the hippocampus. The integrated optical density (IOD) of nNOS was decreased in fluoxetine group (*p* < 0.05 vs. PS group), and markedly decreased in FA groups in a concentration-dependent manner (*p* < 0.05 vs. PS group; Figure 6).

### 2.7. Effects of FA on Cytokine Production

**Figure 7:**
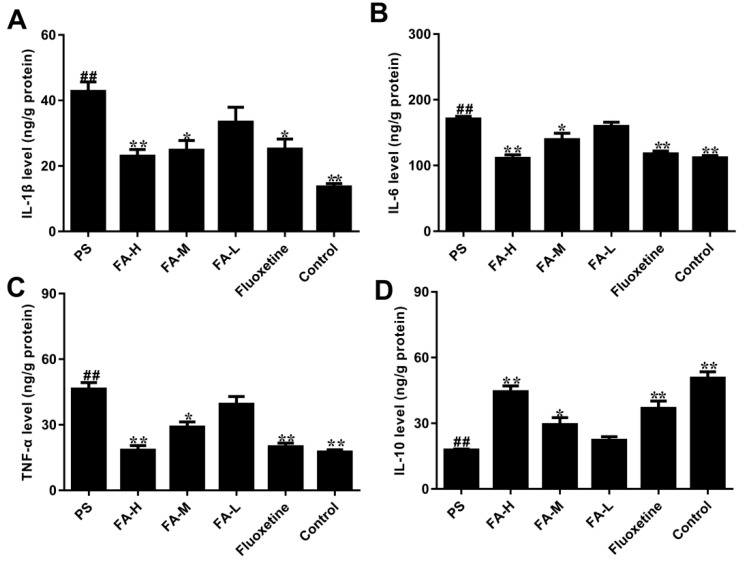
Effects of ferulic acid (FA) on the cytokine concentration in the hippocampus of offspring rats exposed to prenatal stress. (**A**) IL-1β concentration in the hippocampus. (**B**) IL-6 concentration in the hippocampus. (**C**) TNF-α concentration in the hippocampus. (**D**) IL-10 concentration in the hippocampus. Values represent means ± SEM (*n* = 8). * *p* < 0.05 and ** *p* < 0.01 compared with PS group, ^##^ *p* < 0.01 compared with Control group. FA-L: FA at low 12.5 mg/kg/day, FA-M: FA at medium 25 mg/kg/day, FA-H: FA at high 50 mg/kg/day.

As shown in Figure 7, the levels of TNF-α, IL-1β and IL-6 in hippocampus were markedly increased in PS offspring rats when compared to control group (*p* < 0.05), while IL-10 level was significantly decreased after expose to PS. FA treatment can significantly reduce the TNF-α, IL-1β and IL-6 levels, and increase the IL-10 levels in offspring rats expose to PS (*p* < 0.05).

### 2.8. Effects of FA on Cytokine mRNA Expression

**Figure 8:**
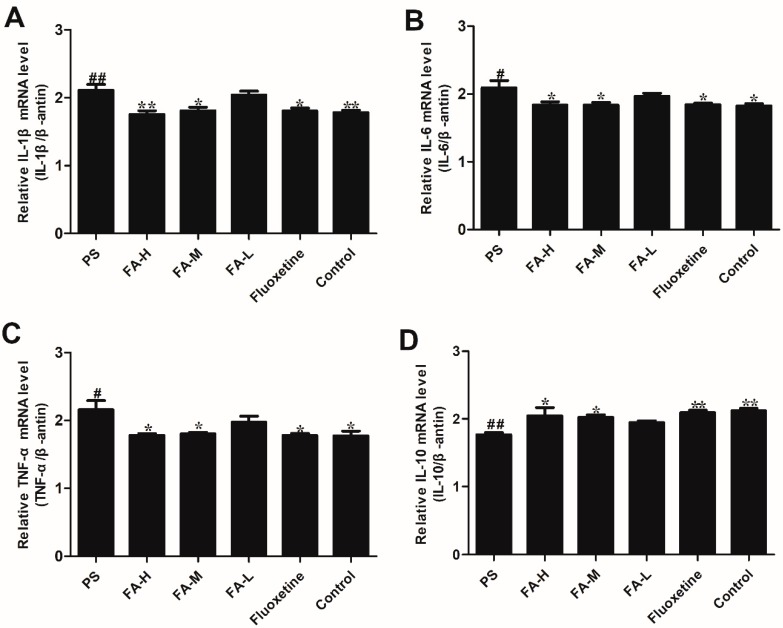
Effects of ferulic acid (FA) on the cytokine mRNA expression in the hippocampus of offspring rats exposed to prenatal stress. Results of (**A**) *IL-1β* mRNA in hippocampus of the offspring. (**B**) *IL-6* mRNA in in the hippocampus. (**C**) *TNF-α* mRNA in the hippocampus. (**D**) *IL-10* mRNA in the hippocampus were quantified by real-time PCR. Values represent means ± SEM (*n* = 3). * *p* < 0.05 and ** *p* < 0.01 compared with PS group, ^#^ *p* < 0.05 and ^##^ *p* < 0.01 compared with Control group. FA-L: FA at low 12.5 mg/kg/day, FA-M: FA at medium 25 mg/kg/day, FA-H: FA at high 50 mg/kg/day.

Consistent with the ELISA results, PS resulted in a significant increase in mRNA expression of *TNF-α*, *IL-1β*, and *IL-6* in the hippocampus in offspring rats when compared with the control rats (*p* < 0.05). In addition, *IL-10* mRNA expression was significantly increased after expose to PS. FA treatment significantly inhibited the PS-induced increase in mRNA expression of *TNF-α*, *IL-1β* and *IL-6*, and caused a reduction of *IL-10* mRNA expression (*p* < 0.05; Figure 8).

### 2.9. Effects of FA on NF-κB Activation and GR Expression

**Figure 9:**
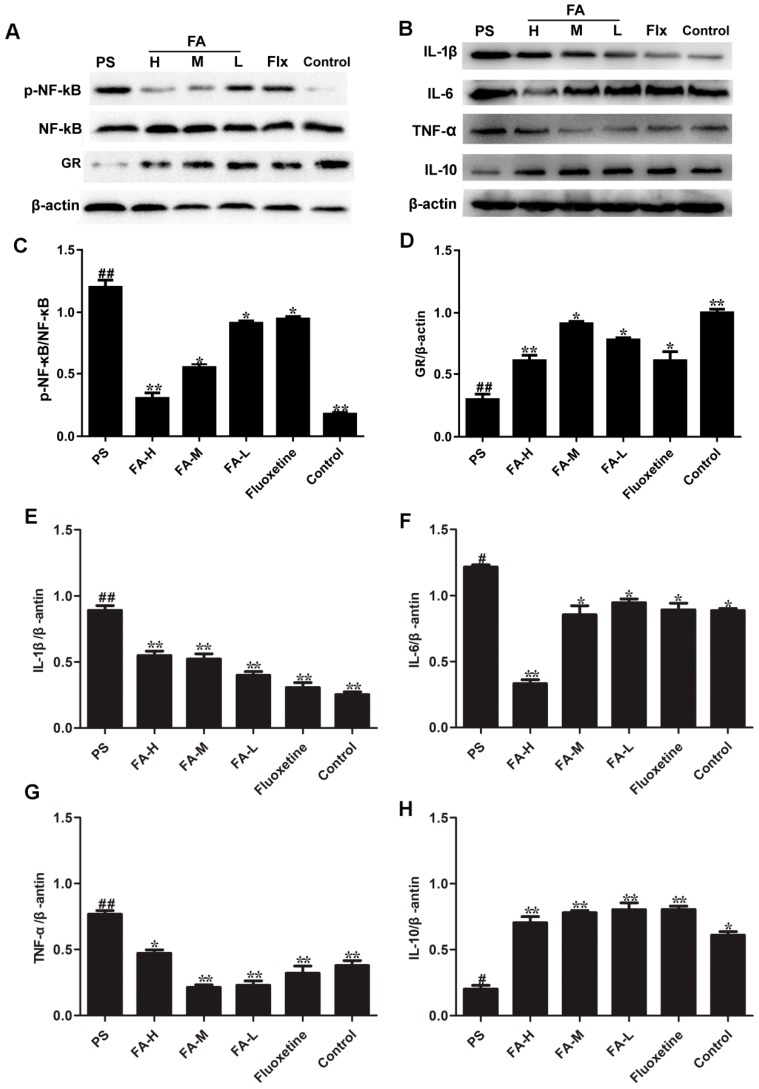
Effects of ferulic acid (FA) on the NF-κB activation, GR and IL family members expression in the hippocampus of offspring rats exposed to prenatal stress. (**A**) Bands of p-NF-κB/NF-κB and GR in the hippocampus. (**B**) Bands of IL family in the hippocampus. (**C**) Bands of p-NF-κB were quantified based on densitometric analysis and then normalization to NF-κB. (**D**) Bands of GR were quantified based on densitometric analysis and then normalization to β-actin protein levels. Bands of (**E**) IL-1β, (**F**) IL-6, (**G**) TNF-α and (**H**) IL-10 were quantified based on densitometric analysis and then normalization to β-actin protein levels. Values represent means ± SEM (*n* = 3). * *p* < 0.05 and ** *p* < 0.01 compared with PS group, ^#^ *p* < 0.01 and ^##^ *p* < 0.01 compared with Control group. FA-L: FA at low 12.5 mg/kg/day, FA-M: FA at medium 25 mg/kg/day, FA-H: FA at high 50 mg/kg/day.

NF-κB is an important upstream modulator of pro-inflammatory cytokines. Thus, we determined whether the FA-induced attenuation in cytokine expression occurred due to a blockade of NF-κB activity. As shown in Figure 9, the chronic treatment of FA markedly suppressed the phosphorylation of NF-κB in the hippocampus in male PS offspring rats.

PS significantly decreased the expression of GR in the hippocampus of offspring rats (Figure 9) compared with the control group (*p* < 0.05). However, FA treatment significantly increased the hippocampal GR protein expression in offspring rats expose to PS (*p* < 0.05). Moreover, effects of FA were similar to fluoxetine group.

In order to explore the influence of FA on IL family protein expression in rat offspring, we measured TNF-α, IL-1β, IL-6 and IL-10 protein expression hippocampus of offspring rats. The results revealed that FA significantly decreased TNF-α, IL-1β and IL-6 protein expression and significantly increased IL-10 protein expression in the hippocampus of offspring rats compared to the PS group (*p* < 0.05, Figure 9).

## 3. Discussion

This study sought to determine the effect of FA on inflammatory mediators and depressive-like behaviors in male offspring rats exposed to PS. We have found that PS-induced anxiety- and depressive-like behaviors were associated with increase in hippocampal inflammatory mediators and HPA axis reactivity. Furthermore, FA administration attenuated the increase in the pro-inflammatory cytokines, IL-1β, IL-6, TNF-α, and decreased IL-10 and depressive-like behavior in PS offspring rats. PS upregulated the hippocampal *IL-1β*, *IL-6*, *TNF-α*, and downregulated the *IL-10* mRNA expression in male offspring rats, which were attenuated by chronic FA treatment. Meanwhile, PS resulted in a marked increase in hippocampal expression of NF-κB and nNOS, which was also attenuated by chronic FA treatment. We also found that FA significantly decreased the ACTH and corticosterone levels in the hippocampus of male PS offspring rats, associated with increased GR expression. These results suggest that the effects of FA may be dependent on its antidepressant and anti-inflammatory effects.

It is well known that the hippocampus is involved in emotional regulation. Many studies have shown that inflammation in this region may lead to depression and anxiety [5,24,25]. Anhedonia has been defined as a decrease in responsiveness to rewards reflected by a reduced intake of palatable sweet solutions, which is a core symptom of human depression. The sucrose preference test has been known to an index of anhedonia-like behavioral alteration [8,26]. In our study, we found that the PS-induced reduction in sucrose preference was significantly reversed by chronic FA treatment, suggesting an anti-depressive-like action of FA.

The early life environment is crucial for individual development. Gestational stress increases maternal circulating GCs. In addition, stress hormones can cross the placental barrier and potentially result in permanent adverse short- and long-term neuroanatomical, biological, behavioral and immune modifications in the offspring [27,28]. GCs can influence the process of maturation and growth of leukocyte, and regulate cytokines include TNF-α, IL-4 and IL-10 secreted by Th1 cells or Th2 cells which modulate cell immune responses and humoral immune responses [8,12]. It has been reported that many cytokines play an important role in stress-related inflammatory disorders and neurological diseases [13]. In IL-6-deficient mice, the results have shown that this cytokine can affect emotional related behaviors, especially anxiety-related behaviors [7]. If cytokines in the brain are dysregulated, they may cause corresponding pathologic changes, including inflammatory, oxidative, and nitrosative molecules and affect neural homeostasis [25]. Increases in serum pro- and anti-inflammatory cytokines have been observed in patients with depression, such as IL-1β, IL-6, and TNF-α, and IL-10, respectively. PS induces alterations in the HPA axis in rats, which can alter immune reactivity [11]. In our study, PS increased the mRNA expression of *IL-1β*, *IL-6*, and *TNF-α* mRNA expression in the hippocampus of male offspring, and decreased *IL-10* mRNA expression, in line with previous studies [7,25]. Chronic treatment with FA dramatically attenuated the PS-induced elevation of pro-inflammatory cytokines. Taken together, these data suggest that the antidepressant-like effects of FA are associated with suppression of pro-inflammatory cytokines.

PS induces the HPA axis reactivity and increases circulating concentration of the stress hormone GCs, and impairs the GR protein in the offspring rats. The hippocampus highly expresses GR, and is particularly vulnerable to GCs manipulations, especially in early life. Prolonged GCs may cause increased activation of excitatory amino acid receptors, including ionotropic glutamate receptors and metabotropic glutamate receptors, and unregulated increases in intracellular Ca^2+^ concentrations, and consequently, increased generation of oxidants, oxidative and inflammatory damage in males [10]. In our study, the hippocampal nNOS expression in offspring rats was measured and found that the nNOS expression in offspring increased significantly in the PS group. According to these results, FA treatment significantly decreased the PS-induced nNOS expression in the hippocampus of male offspring rats. Corticosterone can cross placenta to affect fetal development and induces overproduction of nNOS in PS offspring rats. The overproduction of nNOS in hippocampal neurons therefore indicates that the neurons in the PS group are in a higher oxidative environment, which may gradually cause neuronal injuries. The expression of nNOS increases significantly in the hippocampus of offspring rats exposed to PS rats, indicating that this CA3 region is under higher inflammation and oxidant stress [29]. We also found the neurons decrease significantly in this region. Evidence could help to explain why pyramidal neurons of CA3 are more vulnerable to PS; granule cells of the DG express a high density of GR [11] and these neurons project heavily to CA3 pyramidal cells via mossy fiber output, which might make the neurons of CA3 and DG more vulnerable to the stress levels of GCs. CA3 pyramidal neurons therefore may be particularly vulnerable to PS. Interestingly, in the present study, we found FA decreased serum ACTH and corticosterone levels in offspring rats exposed to PS, suggesting that FA improves depressive-like behavior through the regulation on HPA axis and GR.

NF-κB transcriptionally regulates many cellular genes implicated in early immune, acute phase, and inflammatory responses, inducible NO synthase (iNOS), and pro-inflammatory cytokine. Inhibition of NF-κB activation can suppress the expression of pro-inflammatory cytokines, such as IL-1β, IL-6 and TNF-α [30]. In our study, we demonstrated that FA inhibited the PS-induced activation of NF-κB in the hippocampus of offspring rats, which indicates that the FA-induced inhibition of pro-inflammatory gene expression may dependent on modulation of the NF-κB signaling pathway. FA has shown anti-depressant effects in the tail suspension test in acute depression [31,32], and attenuated chronic neuro inflammation in Alzheimer’s disease [33]. In our study, FA attenuated depressive-like behavior in the male offspring rats exposed PS. The effect is mediated by the NF-κB signaling pathway and GR expression. The results from our present study indicate that FA could be an effective therapeutic treatment in preventing the development of stress-induced, depressive-like behavior in adolescence.

## 4. Materials and Methods 

### 4.1. Animals and Procedures

All animal procedures were carried out in line with the National Institutes of Health Guide for the Care and Use of Laboratory Animals and were ratified by the Institutional Animals Care and Use Committee. All efforts were made to minimize suffering and the number of rats used for the experiments. Male Sprague-Dawley (SD) rats weighing 280–350 g and female SD rats weighing 230–250 g were used for our research. The SD rats were purchased from the experimental animal center of Medical College in Xi’an JiaoTong University. All animals were housed in an animal room with controlled humidity (55 ± 5%) and temperature (25 ± 2 °C) with a 12-h-light/dark cycle (light on from 07:30 to 19:30) with free access to drinking water and food. Virgin female rats and adult male rats (3:1) were housed in one cage for mating; a vaginal smear was tested before 8:00 on the following morning. The day that the smear was sperm positive was defined as embryonic day 0. Thereafter, pregnant rats were housed separately.

Pregnant rats were separately exposed to restraint stress on days 14–20 of pregnancy three times daily for 45 min, as described previously [34]. To prevent the adaptation of animals to the daily procedure, restraint periods were shifted randomly within certain time periods (08:00 A.M.–11:00 A.M., 11:00 A.M.–2:00 P.M., and 4:00 P.M.–7:00 P.M.). A transparent plastic tube (6 cm in diameter) with breathing holes was used as the restraint device. The length of the plastic tube was suited for the size of the animals. Control pregnant rats were not disturbed. All offspring rats were weaned on postnatal day 21, and then separated and housed at no more than four offspring per cage. At postnatal day 60, two female offspring rats from the same biological mother were selected randomly.

### 4.2. Administration of FA in Rats

FA (purity > 98% measured by high-performance liquid chromatography) extracted from *Ligusticum chuanxiong*, was obtained from Shaanxi Huike Botanical Development Co., Ltd (Xi’an, China). Fluoxetine was purchased from Changzhou Siyao Pharmaceuticals Co., Ltd (Changzhou, China). FA and fluoxetine stock solutions were prepared in water containing 0.5% CMC-Na (*m*/*v*). We used a dose–response method to administer, FA at low 12.5 mg/kg/day (FA-L; n = 8), medium 25 mg/kg/day (FA-M; *n* = 8), high 50 mg/kg/day (FA-H; *n* = 8). The, male, prenatally-stressed offspring were treated with 0.5% CMC-Na, and used as the control group. The PS-fluoxetine group was treated with 20 mg/kg/day fluoxetine (*n* = 8). All treatments were administered by the intragastric (i.g.) for 4 weeks.

After 4 weeks of FA and fluoxetine administration, we assessed depressive-like behavior in prenatally-stressed offspring rats using the sucrose preference, forced swimming, and open field tests for 6 days. Following behavioral testing, we removed the hippocampus of all groups. Tissue was stored at −80 °C until processing for ELISA, mRNA extraction or western blotting. The experimental timeline of this study is shown in Figure 1A.

### 4.3. Sucrose Preference Test

Sucrose preference was assessed using a modified version of the sucrose preference test [35] in male, prenatally-stressed offspring rats. The sucrose consumption tests were performed using a two bottles test, with rats having free access to both water and a sucrose solution. Animals were first trained to consume water in the two bottles, and water consumption was measured for 24 h. The next day, sucrose consumption tests began: a bottle filled with a 2% sucrose solution replaced a bottle of water for 4 h. Bottles were counterbalanced across the left and the right sides of the feeding compartment, and alternated in position from test to test. Sucrose preference was calculated as follows: sucrose preference (%) = [sucrose solution intake (mL)/total fluid intake (mL)] × 100.

### 4.4. Forced Swimming Test (FST)

We used the FST to assess depressive-like behavior in the male prenatal stressed offspring rats. The FST was conducted according to previous reports [34]. Rats were forced to swim in a cylindrical tank (height, 50 cm; diameter, 20 cm) filled with water (23–25 °C) to a depth of 40 cm for 8 min. Movements were recorded during the 8-min session by video camera. Immobility time (measured as floating without active forepaws movements) was recorded manually by a trained rater blinded to the status of each rat. Immobility time was calculated as follows: immobility time (%) = [immobility time (s)/total time (s)] × 100. The water in the tank was changed between each test.

### 4.5. Open Field Test (OFT)

We used OFT to assess in the male prenatal stressed offspring. During a 5-min observation period, rats were exposed to an open field (150 × 150 × 49.5 cm; black acrylic walls, green floor), which was divided into a 5 × 5 grid of equally-sized squares using white tape. The central region of the box (3 × 3) was subdivided into a large and a small center of 8 and 1 squares, respectively. The test began by placing each rat on the same side of the small center. Time spent in the center, and the frequency of total crossing counts (total number of squares crossed with all four paws), center crossing counts (total number of center squares crossed with all four paws), rearing counts (standing on hind legs, with or without contact with the sides of the arena), grooming counts (using paws or tongue to clean/scratch body) were recorded. The open field was cleaned with 5% ethanol between each test.

### 4.6. Enzyme-Linked Immunosorbent Assay (ELISA)

After behavioral testing, rats were anaesthetized, then decapitated, and the whole brains were removed quickly. The hippocampus was isolated quickly, then frozen in liquid nitrogen and stored at −80°C for posterior analysis. The concentration of IL-10, TNF-α, IL-6 and IL-1β in the hippocampus were measured using a paired antibody quantitative ELISA kit according to manufacturer’s instructions (KeyGen BioTECH, Nanjing, China).

Meanwhile, blood was collected from the abdominal aorta for corticosterone detection. The blood was centrifuged (3500× g, 15 min) at 4 °C, and the serum was stored at −80 °C until being used for ACTH and corticosterone assays. Corticosterone levels were measured by ELISA according to the instructions of the manufacturer (Shanghai Jianglai Biotech, China).

### 4.7. RNA Isolation and Real-time PCR

Real time RT-PCR was used to detect mRNAs for *IL-1β*, *IL-6*, *IL-10* and *TNF-α*. Total mRNA was isolated from tissues using the Trizol method and quantified by Nano-drop spectrophotometry (Thermo Scientific, MA, America). First strand cDNA was synthesized from 1 μg of each mRNA sample with random primers and a reverse transcription kit (Takara, Kusatsu, Japan). Primer sequences for *IL-1β*, *IL-6*, *IL-10*, *TNF-α*, and *β-actin*mRNA are described in Table 1. SYBR Green real-time PCR Master Mix (Takara, Kusatsu, Japan) was used to detect the abundance of PCR products in the samples. Amplification conditions were: denaturation of all pairs of primers at 94 °C for 30 s; annealing: *IL-1β*, 60 °C for 30 s, *IL-6*, *IL-10* and *TNF-α*, 56 °C for 30 s; elongation: *IL-1β*, *IL-6*, *IL-10* and *TNF-α*, 72 °C for 20 s. Forty cycles were performed. The relative expression of *IL-1β*, *IL-6*, *IL-10* and *TNF-α* mRNA was calculated using the 2^−∆(∆CT)^ comparative method [36], *IL-1β*, *IL-6* and *TNF-α* samples were normalized against the internal endogenous reference, *β-actin*.

### 4.8. Nissl Staining

Animals were sacrificed by cardiac perfusion with 4% paraforaldehyde under anesthesia. The whole brains were removed and processed in paraffin, sectioned at a thickness of 4 μm, and stained with toluidine blue, which are referred to the previous study [10]. For quantitative studies, neurons in the area of CA3 regions were counted using five equally spaced coronal sections passing through the hippocampus for each brain, and quantified by Image-J software.

### 4.9. Immunohistochemical Assay for nNOS

Animals were euthanized and transcardially perfused with 4% paraformaldehyde. The brains were removed, post-fixed in 4% paraformaldehyde for 24 h. The brains were embedded into paraffin and sectioned at a thickness of 4 μm (Leica RM2235, Solms, Germany). Slices were washed with PBS for 5 min × 3 times, then treated with 3% H_2_O_2_ for 10 min. Sections were washed with PBS for 5 min × 3 times, and blocked with 5% BSA for 30 min and incubated in anti-nNOS (1:500) primary antibody diluted in 5% BSA overnight at 4 °C, then further incubated with rat anti-rabbit IgG (30 min, 37 °C). After staining with Mayer’ hematoxylin for nucleus, sections were mounted on poly-L-lysine-coated glass slides, dehydrated, and covered with coverslips by permount tm mounting medium, which can be referred to previous studies [10]. Imaging (400×) was performed using a Nikon Ti-U camera connected to a Nikon Eclipse Timicroscope. The quantified integrated optical density (IOD) of positive nNOS neuron of CA1 region was quantified by Image-J software.

### 4.10. Western Blot Analysis

The protein expression analysis was performed using antibodies against phosphor-NF-κB, GR, IL-1β, IL-6, IL-10 and TNF-α, as described previously [37]. Total hippocampal proteins were electrophoresed using 10% tris-glycine sodium dodecyl sulfate polyacrylamide gels. Then, the gels were transferred to PVDF membranes and blocked using 5% skim milk powder in T-TBS (0.02 M tris/0.15 M NaCl, pH 7.5, and containing 0.1% tween 20) at room temperature for 2 h. After washing three times with T-TBS, the PVDF membranes were incubated with primary antibodies against phosphor-NF-κB and GR (Cell Signaling Technology, MA America), IL-1β, IL-6, IL-10 and TNF-α, (ABclonal, Wuhan, China)overnight at 4 °C. Thereafter, the blots were washed three times with T-TBS and incubated with secondary antibodies (1:4000) at 37 °C for 2 h. β-actin was used as an internal control. Finally, after washing three times with T-TBS, the PVDF membranes were developed using enhanced chemiluminescent (ECL) reagent. The intensity of the bands was analyzed with Image-J software. Intensity values of phosphor-NF-κB were normalized to NF-κB, and GR were normalized to β-actin.

### 4.11. Statistical Analysis

All data are provided as mean ± SEM. Statistical analyses were performed by one-way analysis of variance (ANOVA) comparison using SPSS Version 24.0 (Chicago, IL, USA). *p* < 0.05 was considered statistically significant.

## Figures and Tables

**Table 1:** Primers used for IL-1β, IL-6, IL-10, TNF-α and GAPDH.

Primers	Forward/ Reverse	Sequence
IL-1β	Forward	5′-aatgcctcgtgctgctg-3′
	Reverse	5′-tgtcgttgcttgtctctcc-3′
IL-6	Forward	5′-ccagagtcattcagagcaatac-3′
	Reverse	5′-gatggtcttggtccttagcc-3′
IL-10	Forward	5′-tgccttcagtcaagtgaagact-3′
	Reverse	5′-aaactcattcatgccttgta-3′
TNF-α	Forward	5′-ccacgctcttctgtctactg-3′
	Reverse	5′-ctacgggcttgtcactcg-3′
β-actin	Forward	5′-tacaaccttcttgcagctcctc-3
	Reverse	5′-gccgtgttcaatggggtact-3′
